# Mitochondrial divergence suggests unexpected high species diversity in the opsariichthine fishes (Teleostei: Cyprinidae) and the revalidation of *Opsariichthys macrolepis*

**DOI:** 10.1002/ece3.4933

**Published:** 2019-02-05

**Authors:** Xue Wang, Fei Liu, Dan Yu, Huanzhang Liu

**Affiliations:** ^1^ The Key Laboratory of Aquatic Biodiversity and Conservation of Chinese Academy of Sciences, Institute of Hydrobiology Chinese Academy of Sciences Wuhan China; ^2^ University of Chinese Academy of Sciences Beijing China

**Keywords:** cryptic species, cytochrome *b*, opsariichthine, *Opsariichthys macrolepis*, species delimitation, the upper Yangtze River

## Abstract

Opsariichthine (sensu *Oceanologi Et Limnologia Sinica*, 1982, 13, 293–298) is a cyprinid group consisting of five genera and endemic to East Asia. Previous studies suggested that there may be many possible cryptic species in this group, but this has not been confirmed. In this study, using mitochondrial cyt *b* sequences on 1,388 samples and 739 haplotypes, we showed very high species diversity within this group. The results showed that phylogenetic relationships of the opsariichthine group were as ([*Nipponocypris*‐*Parazacco*‐*Candidia*] + [*Zacco* + *Opsariichthys*]), and there were multiple deep lineages within several species, flagging putative cryptic species. When a 3% genetic distance was used as a threshold for species delimitation, 35 haplogroups were found, nine haplogroups in *Candidia*‐*Parazacco*‐*Nipponocypris* group, six haplogroups in the *Zacco *group, and 20 haplogroups in the *Opsariichthys* group. We consider all of them to be putative until determination of distinct species based on the tree topology, geographic distributions, or a combination of both. In addition, two kinds of species delimitation tools, ABGD and PTP, were applied to construct molecular operational taxonomic units (MOTUs). The ABGD method revealed nine MOTUs in *Candidia*‐*Parazacco*‐*Nipponocypris* group, two MOTUs in the *Zacco *group, and 17 MOTUs in the *Opsariichthys* group. And the PTP method revealed 10 MOTUs in *Candidia*‐*Parazacco*‐*Nipponocypris* group, 10 MOTUs in the *Zacco *group, and 29 MOTUs in the *Opsariichthys* group. Therefore, there should be more species in the opsariichthine group than presently described. Based on the molecular data and morphological characteristics, we proposed *Opsariichthys macrolepis* as a valid species and described its morphological diagnostic characters.

## 1 INTRODUCTION

The identification and delimitation of species, one of the main objectives of taxonomy, are important in evolutionary biology because species remain the fundamental unit and operational entity in most disciplines (Durand & Borsa, 2015). Taxonomic descriptions of species are typically accomplished through morphological criteria that were established by earlier typological studies. However, misidentification often occurred because of features such as phenotypic plasticity, cryptic species, genotypic variation, or different life history stages (species that exhibits polymorphism, e.g., sexual dimorphism and mimicry; Chen, Ma, Shen, Mao, & He, 2015). They could lead to erroneous estimate of genetic diversity, genetic differentiation between populations, the risks of local extinction, or producing meaningless estimates of demographic parameters and in turn may misguide management actions (Durand & Borsa, 2015). While deciphering hidden diversity in species remains a taxonomic challenge, it is important to study the species differentiation and to understand patterns and processes in biodiversity (Butlin, Bridle, & Schluter, 2009).

DNA barcoding has been proposed as a quick and inexpensive approach to species identification, species discovery, and species delimitation. The mtDNA such as cytochrome oxidase 1 (CO1) or cytochrome *b *(cyt *b*) is usually employed as the universal locus (e.g., Hanelt, Schmidt‐Rhaesa, & Bolek, 2015; Hundt, Berendzen, & Simons, 2017; Kakioka et al., 2018), and it is easier to amplify from highly processed and degraded tissues than nuclear DNA (Yang & Rannala, 2017). Molecular studies have been crucial to improve our knowledge on the ichthyofauna, and DNA barcoding has been successfully used in fish species identification and in detecting species of taxonomic concerns or cryptic diversity (Gomes, Pessali, Sales, Pompeu, & Carvalho, 2015; Pereira, Hanner, Foresti, & Oliveira, 2013). For example, Ramirez et al. (2017) using a DNA barcoding approach detected hidden biodiversity within a recently described freshwater fish genus *Megaleporinus* and identified 16–18 different molecular operational taxonomic units (MOTUs) within each of the 10 studied nominal species. For the genus *Salminus*, which was once migratory fish and top predator distributed throughout South America major hydrographic basins, Machado, Ishizuka, Freitas, Valiati, and Galetti (2016) employed the standard DNA barcoding analyses and DNA delimitation approaches, and determined eight MOUTs including the four nominal species. Based on the results, they suggested a new taxonomic scenario and conservation policy for *Salminus* in Brazil.

The Cyprinidae (cyprinids) represent one of the most diverse freshwater fish groups and are the major components of the primary freshwater fish fauna in Africa, Eurasia, and North America, comprising more than 367 genera and 3,006 species (Nelson, Grande, & Wilson, 2016). The opsariichthine fishes are one of the East Asian endemic minnow group of cyprinids and comprise five genera, *Candidia *(Jordan & Richardson, 1990), *Nipponocypris* (Chen, Wu, & Hsu, 2008), *Opsariichthys* (Bleeker, 1863), *Parazacco* (Chen, 1982), and *Zacco* (Jordan & Evermann, 1902). Opsariichthine have been taxonomically defined by Chen (1982) as a group of minnows in Cyprinidae sensu lato occurring widely in East Asia, with the large and elongate anal fin and a series of nuptial tubercles on the jaws as common features in adults (Chen, 1982). This group included small‐sized fish that prefer to live in rivers or streams and swim actively in riffles with swiftly running waters (Chen & Chang, 2005; Chen, 1982; Shen & Tzeng, 1993). Among these, *Nipponocypris* is distributed in the Korean Peninsula and Japan mainly. *Candidia* and *Parazacco *are confined to Chinese Taiwan and southeastern continental Asia, respectively. The remaining two genera, *Zacco* and *Opsariichthys*, are widely distributed in East Asia.

For more than one hundred years, taxonomic descriptions of opsariichthine species were largely accomplished through morphological characteristics alone. However, recent molecular biological approaches revealed a complicated scenario. A series of reports on the taxonomy of opsariichthine based on morphological and genetic analyses was published, and the reports proposed the new classification of these Asian minnows (Chen & Chang, 2005; Chen, Huang, Jang‐Liaw, Shen, & Wu, 2008; Chen, Wu, & Huang, 2009; Huynh & Chen, 2013). Such as, (a) three new species were identified, *Opsariithchys duchuunguyeni* from northern Vietnam, *Opsariithchys kaopingensis* and *Candidia pingtungensis* from southern Taiwan, (b) *Zacco evolans* from southern China and Taiwan, *Zacco acutipinnis* from southern China, and *Zacco pachycephalus *from Taiwan were suggested as members of *Opsariichthys*, (c) *Opsariichthys minutus* from central and southern China and *Opsariichthys hainanenisi* from Hainan were considered as valid, (d) *Opsariichthys heini* and *Opsariichthys bea *were in fact unrelated to *Opsariichthys*, but closely related to genus *Rasbora* and *Parazacco, *respectively, by morphological description.

Recent studies also indicated that there is a hidden diversity within *O*. *bidens* and *Z*. *platypus *(Berrebi, Boissin, Fang, & Cattaneo‐Berrebi, 2005; Perdices & Coelho, 2006; Perdices, Cunha, & Coelho, 2004; Perdices, Sayanda, & Coelho, 2005). *O. bidens* lives in sympatry with *Z. platypus* in many localities, and they are considered widespread species in the Chinese Mainland. However, the population genetic studies found higher genetic structure and long‐term interruption of gene flow than previously expected (Berrebi et al., 2005; Perdices & Coelho, 2006; Perdices et al., 2004, 2005). In these studies, four *Z. platypus* and five *O. bidens* mtDNA lineages were resolved and suggested to correspond to four and five species, respectively. Supporting the idea, Johansson (2006) examined body shape differences among the mtDNA lineages, which were based on results of Perdices et al. (2004), Perdices et al. (2005) and found that the different lineages were reflected in body shape differences. Similar results were also found by Berrebi et al. (2005) and Li, Wang, Zhao, Zhang, and Zhang (2009). They suggested the possibility of more species existing in the opsariichthine group.

Although these authors realized that many cryptic species exist in the opsariichthine group, they have not made any progress in taxonomy except the works by Chen, Huang et al. (2008), Chen, Wu et al. (2008), Chen et al. (2009) and Huynh and Chen (2013). In this paper, we used molecular approach to conduct a comprehensive investigation of the opsariichthine group, assessing all nominal species and lineages previously described. Our objective is to provide a clear phylogenetic structure to support identification and designation of species in opsariichthine through the use of several approaches in DNA taxonomy and to revise the current nomenclature of species by proposing new, provisional names to these lineages. Based on molecular analysis, we found opsariichthine from the upper Yangtze River should be a separate species. We compared the morphological characters of 30 specimens from the upper Yangtze River and found their morphological characters are in congruent with the *Opsariichthys macrolepis*. Therefore, we discussed the validation of *Opsariichthys macrolepis*. In doing so, we hope to make a little progress on the taxonomy of this speciose group.

## 2 MATERIAL AND METHODS

### 2.1 Sampling

**Figure 1:**
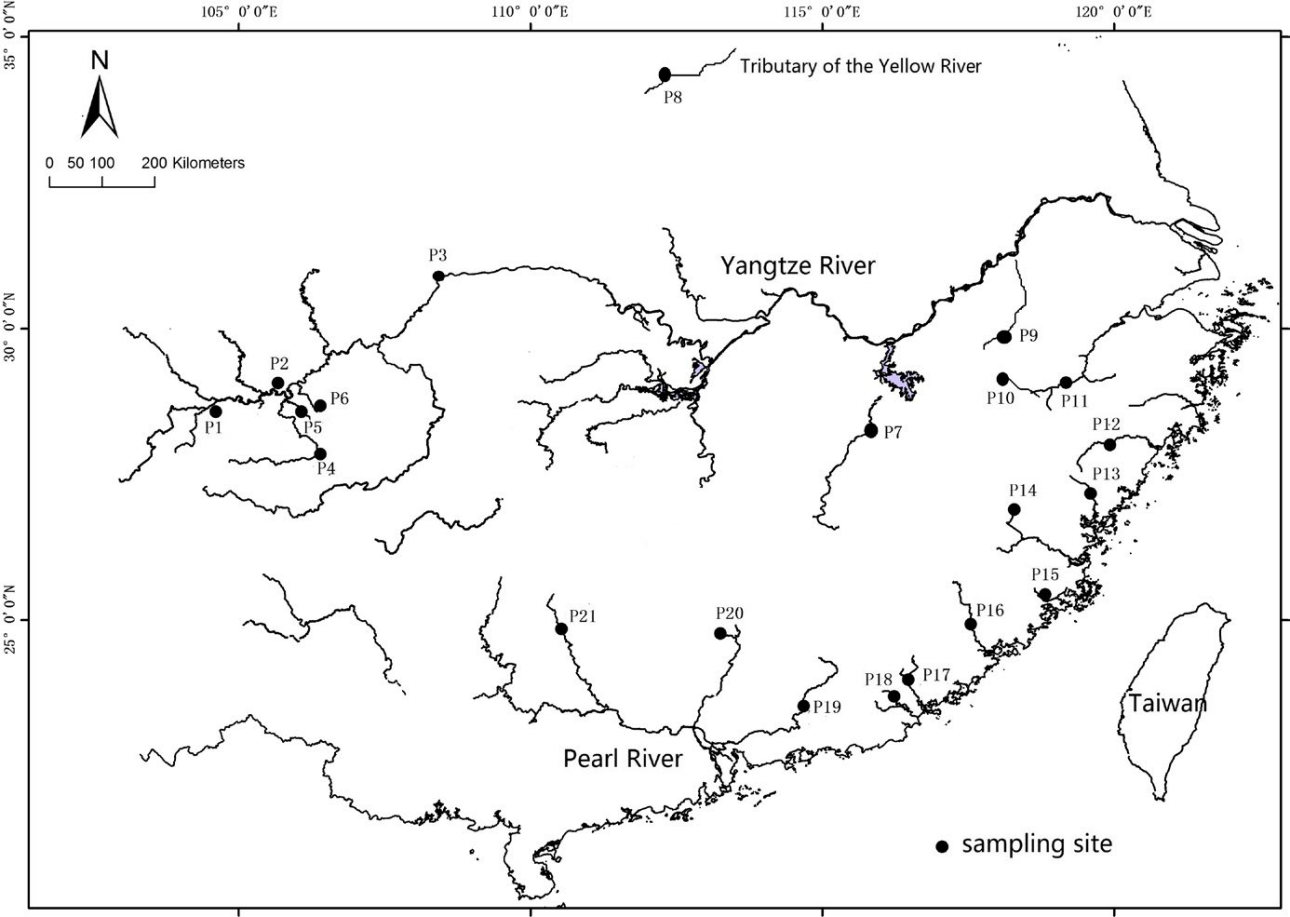
Collection sites for the newly generated sequences of the present study. Details of the 21 sites and collected specimens are provided in Supporting Information Table S1. The numbers provided are the sample site numbers. This map was created in the ArcGIS version 10.1 (http://www.esri.com/arcgis/about-arcgis)

From 2010 to 2017, 371 individuals from the opsariichthine group were collected from various localities in the Yangtze River, Huang River, Pearl River, and the freshwaters of the southeast coastal areas of China by our research group (Supporting Information Table S1; Figure 1). Specimens were identified following the diagnostic characters described by Bǎnǎrescu (1968), Chen (1982), Chen and Chu (1998), and FISHBASE (Froese & Pauly, 2018); the latter database was used whenever additional morphological diagnostic characters were described online. The samples for DNA extraction were preserved in 95% ethanol, and for morphological analysis, specimens were fixed in 10% formalin. A set of reference individuals was deposited in the Institute of Hydrobiology, Chinese Academy of Sciences.

### 2.2 Laboratory methods

Total DNA was extracted from muscle tissue with a standard salt extraction protocol of Aljanabi and Martinez (1997). The cytochrome *b* gene was amplified using a polymerase chain reaction (PCR) with primers CGlu‐2 (AACCACCGTTGTAATTCAACTA) and Pro‐R1 (TAGTTTAGTTTAGAATTCTGGCTTTGG) adopted from Hardman and Page (2003) for the samples from Qingyi River in Huangshan City, Anhui Province, and for the rest of the samples, we used the primers L14724 (5ʹ‐GACTTGAAAAACCACCGTTG‐3ʹ) and H15915 (5ʹ‐CTCCGATCTCCGGATTACAAGAC‐3ʹ) by Xiao, Zhang, and Liu (2001). Each 50 μl PCR reaction contained 100 ng of template DNA, 1 μl of each primer (each 10 μM), 5 μl of 10× reaction buffer, 2 μl dNTPs (each 2.5 mM), and 2.0 U Taq DNA polymerase. The reactions were performed as following: an initial 94°C denaturation for 4 min, and then, 35 cycles of 94°C denaturation for 45 s, 56°C annealing for 45 s, 72°C extension for 1 min, and a final 72°C extension for 10 min. Amplified DNA was fractionated by electrophoresis through 0.8% agarose gels, recovered from the gels, and purified using the BioStar glassmilk DNA purification kit following the manufacturers’ instructions. The same primers CGlu‐2/Pro‐R1 and L14724/H15915 were used for sequencing. Bidirectional sequencing was employed to decrease the occurrence of sequencing error by commercial companies. The sequences have been deposited in GenBank (accession numbers are listed in Supporting Information Table S1).

### 2.3 Phylogenetic analyses and genetic distance

In this study, a total of 1,388 sequences were employed. 371 sequences were newly sequenced and 1,017 sequences were obtained from GenBank, which were primarily from Perdices et al. (2004), Perdices et al. (2005), Perdices and Coelho (2006), Li et al. (2009), Lin et al. (2016), and Kitanishi et al. (2016), with additional data drawn from other relevant publications (Takamura & Nakahara, 2015; Wang, Hsieh, Lee, & Wang, 2011; Wang, Wang, Du, & Lee, 2007; Xi, Li, Wang, Nie, & Xie, 2016; Yin, Cao, He, & Fu, 2015; Zheng et al., 2016). A full list of sequences with corresponding GenBank accession numbers along with sampling localities is provided in Supporting Information Table S1.

Sequences were aligned using Clustal X (Thompson, Gibson, Plewniak, Jeanmougin, & Higgins, 1997) and refined manually with SEAVIEW (Galtier & Gouy, 1996). Sequence variations such as nucleotide composition, variable sites, and parsimony informative site were calculated with MEGA 5.0 (Tamura et al., 2011). Phylogenetic analyses were performed with haplotype data, which were collapsed using ALTER (Glezpeña, Gómezblanco, Reboirojato, Fdezriverola, & Posada, 2010). For outgroup, we selected three nonopsariichthine cyprinids, *Aphyocypris chinensis *(AF307452), *Aphyocypris kikuchii *(JX184925), and *Yaoshanicus arcus *(AF309086).

Based on the cyt *b* gene, phylogenetic relationships among the opsariichthine haplotypes were reconstructed using three methods, Neighbor‐joining (NJ), Bayesian inference (BI), and maximum likelihood (ML). The best‐fit model of nucleotide evolution for the data was identified by Modeltest 3.7.0 (Posada & Crandall, 1998). NJ analysis was performed with MEGA 5.0 (Tamura et al., 2011) using the Kimura's 2‐parameter (K2P) model. Bootstrapping with 1,000 pseudo replicates was used to examine the robustness of clades in the resulting tree (Felsenstein, 1985). BI was performed with MrBayes 3.2.2 (Huelsenbeck & Ronquist, 2001). The TrN+I+G substitution model (I = 0.5245, G = 1.3349) was selected based on Bayesian information criterion (BIC). In BI, two independent analyses with four simultaneous Makov chains Monte Carlo (MCMC) runs of 6,000,000 generations were made sampling every 1,000 generations, three heated chains and one cold chain, with a total 6,001 trees each. After testing for convergence of the MCMC algorithm, the first 2,000 trees were discarded as burnin. A 50% majority rule consensus tree was obtained from the remaining 4,001 trees. Posterior probabilities (PP) of phylogenetic inferences were determined from remaining trees. The ML method was performed with RAxML v.8.1.21 (Stamatakis, 2014). The best scoring ML tree was identified using a nucleotide substitution model of GTR+I+G. The support of each node was estimated using a rapid bootstrap analysis with 1,000 replicates.

Genetic divergence is generally measured by the estimated pairwise distance between sequences, such as the p‐distance, or more commonly, the distance calculated by the Kimura's 2‐parameter (K2P) model (Kimura, 1980; Kondo, Ueno, Ohbayashi, Golygina, & Takamura, 2016). In this study, genetic distance was calculated with MEGA 5.0 (Tamura et al., 2011) with the default parameters and 1,000 bootstrap replicates under K2P model. The early studies proposed that levels of neutral mitochondrial DNA sequence diversity play a prominent role in alpha taxonomy, with divergence thresholds of approximately 3% widely being accepted as indicative of species differences (Chappell, Trewick, & Morganrichards, 2011; Hebert, Cywinska, Ball, & DeWaard, 2003; Johnston, Morikawa, Ntie, & Anthony, 2011). In this study, we reviewed all case in which haplogroups separated from the closest neighboring haplogroup by a nucleotide distance larger than the threshold (3%) and considered them to potentially represent additional cryptic species. For these cryptic species, we used Eschmeyer's (2018) fish database as the reference for the current nomenclature (Durand & Borsa, 2015). The current nomenclature was maintained for a haplogroup when its geographic distribution was compatible with the type locality of the species. We maintained the current nomenclature to designate those haplogroups that unambiguously correspond to the type material, based on the type locality, and we arbitrarily assigned capital letters or Arabic numbers to the other haplogroups. The other haplogroups were thus provisionally denominated “sp. A,” “sp. B,” etc.

### 2.4 DNA taxonomy

In this study, to group samples into MOTUs and further delimit species, two popular approaches, the Automatic Barcode Gap Discovery (ABGD) and The Poisson Tree Process (PTP), were applied.

In most situations, genetic distances between individuals from different species are supposed to be greater than the intraspecific variation, revealing a noncontinuous distribution (Hebert et al., 2003). This feature is called a barcode gap, which can be used as a threshold offering primary species delimitation under the assumption that individuals within a species are more similar than between species (Mallet, 1995). ABGD is an automatic procedure that can directly sort sequences into putative species based on barcode gaps (Puillandre, Lambert, Brouillet, & Achaz, 2012). ABGD was performed on a web interface (wwwabi.snv.jussieu.fr/public/abgd) using the default values for the relative gap width (*X* = 1.5) and two distance metrics (JC69 and K2P) as well as the p‐distance (Puillandre et al., 2012).

PTP is a model for delimiting species on a phylogenetic tree by searching for sequence clusters, thus distinguishing within and between species branching (Zhang, Kapli, Pavlidis, & Stamatakis, 2013). A ML tree generated with RAxML v.8.1.21 (Stamatakis, 2014) based on 1,000 rapid bootstrap replicates under the GTR+I+G model was used as an input data. The PTP analysis was conducted on the PTP web server (http://species.h-its.org/ptp/), using default options and 500,000 MCMC generations. PTP reports were generated using the maximum likelihood (ML) tree.

In DNA taxonomy analysis, in order to avoid ambiguous alignments, three sequence datasets (the *Candidia*‐*Parazacco*‐*Nipponocypris* complex group, the genus *Zacco* group, and the genus *Opsariichthys* group) representing species complexes or genera based on the phylogeny and morphological characterization were selected and the analysis was run on each of the datasets. In case of discordance in the amount of splitting, we chose to keep the smallest number of entities, in order to avoid over splitting the species.

### 2.5 Morphological studies

Based on the gene tree and the results of the DNA taxonomy, the samples from the upper Yangtze River were suggested as a separate species named *Opsariichthys macrolepis*. In order to accurately identify it, we examined 30 specimens (83.78–120.33 mm SL) collected from the upper Yangtze River which were IHCAS XSR‐15032380‐2392, 94.30–119.56 mm SL, Chishui City, and IHCAS CSR‐20160424001‐017, 83.78–120.33 mm SL, Renhuai City (Supporting Information Table S2).

Methods for measurement followed Chen and Chang (2005), Chen et al. (2009), and Huynh and Chen (2013). Morphometric characters were measured with digital calipers and recorded to the nearest 0.1 mm. Counts and measurements were made on the left side whenever possible. The measurements included standard length (SL), body depth (BD), eye length (EL), head length (HL), caudal peduncle length (CPL), snout length (SNL), interorbital width (IOW), lateral line scales, scales below lateral line, scales above lateral line, predorsal scales, scales surrounding caudal peduncle, gill rakers, and the number of rows of pharyngeal. Five meristic characters were recorded including the number of branched rays of dorsal (D), pectoral (P1), pelvic (P2), and anal (A) fins. Some terms used to describe the nuptial tubercles arranged on the jaw were based on the woks of Huynh and Chen (2013).

Huynh and Chen (2013) compared the species in genus *Opsariichthys* by morphometric characters and raised a series of diagnostic key features. In this paper, morphological variation among the revalidated species and other congeners which were described in Huynh and Chen (2013) such as *O. acutipinnis* and *O. evolans* were discussed further.

## 3 RESULTS

### 3.1 Mitochondrial haplogroups and the cryptic species in the opsariichthine fishes

A total of 371 sequences of the complete cytochrome *b *gene (1,140 bp) consisted of five opsariichthine species (*O. acutipinnis*, *O. bidens*, *O. evolans*, *Z. platypus*, and *Z. acanthogenys*) were new sequences in this study, and 1,017 sequences were supplemented from GenBank consisted of 18 opsariichthine species (*C. barbatus*, *C. pingtungensis*, *N*. *sitboldii*, *N. temminckii*, *P. fasciatus*, *P. spilurus*, *O. acutipinnis*, *O. bidens*, *O. chengtu*i, *O. duchuunguyeni*, *O. evolans*, *O. hainanensis*, *O. kaopingensis*, *O. minutes*, *O. pachycephalus*, *O. uncirostris*, *Z. acanthogenys*, *and Z. platypus*). Due to length heterogeneities of the cyt *b* sequences from NCBI, the aligned data matrix used for the analyses consisted of 913 bp sequence for cyt *b* in the present study. In total, 739 unique haplotypes were identified from 1,388 sequences. The number of mutations was 423 and the number of parsimony informative sites was 378. The base frequencies were A = 24.7%, C = 28.2%, G = 16.4%, and T = 30.7%. The sequence character analysis indicated that the G‐C frequency (44.6%) was apparently lower than the A‐T frequency (55.4%), which is consistent with the features of the mitochondrial genome (Zhang & Hewitt, 1996).

**Figure 2:**
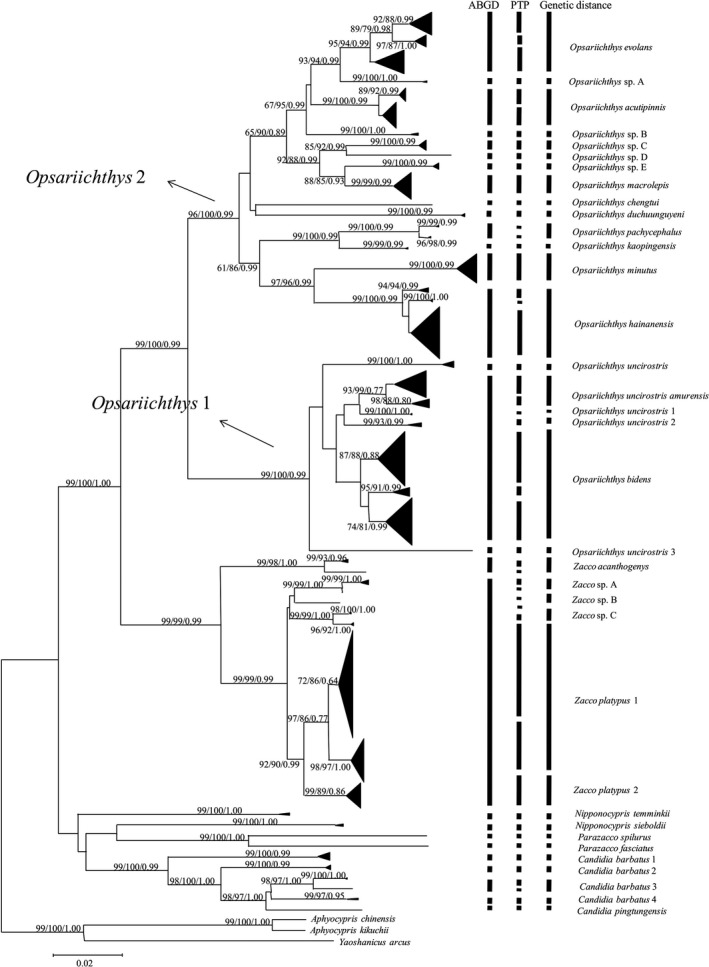
Neighbor‐joining tree of opsariichthine showing the clustering of the MOTUs obtained by the species delimitation analyses based on cytochrome *b *gene sequences. Values at the nodes correspond to the support values for Neighbor‐joining, maximum likelihood and Bayesian inference (NJ/ML/BI) methods

ML, NJ, and BI analyses generated similar tree topologies, and the NJ tree was shown in Figure 2. The molecular phylogenetic relationships show that the opsariichthine comprises three main groups: the *Candidia*‐*Parazacco*‐*Nipponocypris* group with one longitudinal stripe on the flanks, the *Zacco *group with an indistinct vertical stripe or band on the side of the body, and the *Opsariichthys* group with several distinct vertical stripes or bands along its body. The result showed that, when a 3% genetic distance was used as a threshold for species delimitation, there were 35 haplogroups in the mitochondrial phylogeny of the opsariichthine fishes, nine haplogroups in *Candidia*‐*Parazacco*‐*Nipponocypris* group, six haplogroups in the *Zacco *group, and 20 haplogroups in the *Opsariichthys* group. We consider all of them to be putative, distinct species. These cases are examined genus by genus in the following analysis, where each haplogroup was either assigned a capital letter, Arabic numbers, or its current name. Pairwise distributions of nucleotide distance among and within each of the 35 haplogroups were calculated with the Kimura 2‐parameter model. Genetic distances between haplogroups ranged from 3.3% to 20.9% (Table 1).

**Table 1:** Nucleotide distance between haplogroups under the K2P model

	1	2	3	4	5	6	7	8	9	10	11	12	13	14	15	16	17	18	19	20	21	22	23	24	25	26	27	28	29	30	31	32	33	34	35
1 *C.barbatus* 1																																			
2 *C.barbatus *2	0.089																																		
3 *C.barbatus *3	0.088	0.066																																	
4 *C.barbatus *4	0.090	0.072	0.045																																
5 *C.pingtungensis*	0.090	0.064	0.047	0.055																															
6 *N.temminckii*	0.118	0.126	0.132	0.132	0.135																														
7 *N.sieboldii*	0.122	0.125	0.126	0.133	0.133	0.113																													
8 *P.spilurus*	0.149	0.160	0.157	0.150	0.159	0.141	0.136																												
9 *P.fasciatus*	0.154	0.156	0.164	0.162	0.160	0.156	0.141	0.095																											
10 *Zacco* sp. C	0.150	0.153	0.161	0.158	0.162	0.143	0.157	0.171	0.174																										
11 *Zacco* sp. A	0.154	0.151	0.155	0.152	0.155	0.142	0.149	0.167	0.167	0.037																									
12 *Z.platypus *1	0.145	0.150	0.152	0.151	0.150	0.138	0.148	0.167	0.170	0.038	0.043																								
13 *Z.platypus *2	0.144	0.141	0.151	0.152	0.151	0.128	0.148	0.169	0.169	0.040	0.043	0.032																							
14 *Z.acanthogenys*	0.143	0.149	0.147	0.145	0.156	0.136	0.145	0.163	0.176	0.068	0.074	0.074	0.072																						
15 *O.uncirostris amure*	0.181	0.179	0.170	0.177	0.176	0.160	0.162	0.175	0.180	0.143	0.139	0.141	0.136	0.142																					
16 *O.uncirostris *1	0.174	0.168	0.163	0.168	0.171	0.158	0.169	0.172	0.184	0.139	0.141	0.135	0.133	0.140	0.034																				
17 *O.uncirostris* 2	0.176	0.172	0.168	0.172	0.174	0.157	0.161	0.173	0.181	0.139	0.142	0.140	0.136	0.138	0.041	0.043																			
18 *O.bidens*	0.166	0.168	0.166	0.171	0.173	0.163	0.162	0.181	0.179	0.137	0.139	0.135	0.132	0.138	0.040	0.046	0.037																		
19 *O.minutus*	0.166	0.168	0.171	0.169	0.174	0.169	0.181	0.180	0.188	0.143	0.145	0.147	0.149	0.150	0.144	0.143	0.129	0.132																	
20 *O.uncirostris*	0.189	0.182	0.176	0.183	0.177	0.164	0.171	0.187	0.185	0.147	0.152	0.146	0.145	0.147	0.056	0.056	0.060	0.065	0.139																
21 *O.uncirostris *3	0.172	0.181	0.168	0.171	0.169	0.163	0.172	0.180	0.188	0.148	0.151	0.150	0.149	0.147	0.076	0.074	0.079	0.077	0.147	0.080															
22 *O.pachycephalus*	0.168	0.159	0.170	0.172	0.171	0.162	0.167	0.183	0.185	0.138	0.142	0.140	0.137	0.140	0.125	0.128	0.115	0.122	0.094	0.130	0.125														
23 *O.kaopingensis*	0.172	0.158	0.170	0.171	0.175	0.156	0.165	0.187	0.173	0.139	0.142	0.136	0.134	0.147	0.114	0.119	0.107	0.112	0.094	0.118	0.126	0.046													
24 *O.hainanensis*	0.162	0.159	0.160	0.166	0.175	0.157	0.153	0.169	0.172	0.143	0.141	0.145	0.141	0.145	0.127	0.126	0.125	0.122	0.078	0.133	0.138	0.097	0.097												
25 *O.evolans*	0.161	0.163	0.173	0.171	0.171	0.155	0.167	0.185	0.176	0.141	0.141	0.139	0.136	0.145	0.126	0.123	0.121	0.116	0.095	0.130	0.123	0.095	0.086	0.093											
26 *Opsariichthys *sp. A	0.161	0.161	0.167	0.163	0.170	0.155	0.159	0.175	0.170	0.141	0.135	0.140	0.135	0.148	0.127	0.127	0.125	0.118	0.101	0.136	0.128	0.097	0.085	0.091	0.046										
27 *O.acutipinnis*	0.159	0.165	0.174	0.174	0.176	0.163	0.171	0.181	0.177	0.130	0.131	0.130	0.128	0.145	0.124	0.116	0.117	0.115	0.101	0.127	0.118	0.104	0.088	0.101	0.052	0.058									
28 *Opsariichthys *sp. B	0.147	0.156	0.166	0.169	0.167	0.160	0.162	0.169	0.173	0.140	0.140	0.140	0.136	0.145	0.125	0.121	0.120	0.115	0.098	0.137	0.120	0.095	0.091	0.093	0.058	0.064	0.058								
29 *Opsariichthys *sp. E	0.164	0.161	0.175	0.177	0.167	0.164	0.162	0.185	0.179	0.146	0.142	0.146	0.142	0.144	0.125	0.124	0.124	0.117	0.102	0.135	0.127	0.102	0.094	0.102	0.073	0.087	0.077	0.068							
30 *O.macrolepis*	0.165	0.154	0.172	0.176	0.166	0.163	0.166	0.180	0.183	0.135	0.135	0.133	0.126	0.141	0.125	0.121	0.121	0.118	0.105	0.131	0.131	0.098	0.085	0.103	0.072	0.077	0.068	0.068	0.044						
31 *Opsariichthys *sp. C	0.158	0.157	0.167	0.170	0.168	0.162	0.170	0.186	0.190	0.145	0.146	0.138	0.136	0.140	0.129	0.125	0.125	0.117	0.099	0.138	0.121	0.097	0.091	0.101	0.076	0.083	0.077	0.074	0.067	0.061					
32 *O.duchuunguyeni*	0.181	0.192	0.187	0.188	0.191	0.176	0.182	0.209	0.198	0.149	0.153	0.150	0.151	0.160	0.149	0.148	0.138	0.140	0.124	0.152	0.143	0.124	0.126	0.123	0.112	0.115	0.109	0.102	0.101	0.105	0.104				
33 *O.chengtui*	0.160	0.171	0.174	0.175	0.180	0.158	0.162	0.183	0.176	0.141	0.142	0.136	0.141	0.148	0.149	0.143	0.141	0.142	0.105	0.145	0.143	0.093	0.095	0.104	0.096	0.107	0.100	0.082	0.089	0.092	0.089	0.102			
34 *Zacco *sp. B	0.146	0.144	0.151	0.149	0.149	0.131	0.151	0.161	0.170	0.035	0.032	0.035	0.038	0.065	0.135	0.129	0.130	0.138	0.145	0.142	0.146	0.137	0.139	0.144	0.139	0.136	0.131	0.140	0.146	0.136	0.146	0.157	0.143		
35 *Opsariichthys *sp. D	0.153	0.153	0.159	0.162	0.157	0.155	0.163	0.180	0.179	0.148	0.146	0.143	0.140	0.146	0.135	0.133	0.126	0.127	0.104	0.142	0.136	0.099	0.092	0.102	0.081	0.085	0.082	0.072	0.066	0.055	0.052	0.105	0.099	0.148	

In the genus *Candidia*, 21 haplotypes were grouped into five deeply rooted haplogroups that apparently have distributions in Chinese Taiwan and these haplogroups showed 4.5%–8.9% nucleotide divergence under the K2P model. Four haplogroups identified as *C. barbatus* were paraphyletic with *C. pingtungensis*. Since these four haplogroups may represent different species, they were labeled as *C. barbatus* 1–4. In the genus *Parazacco*, two haplogroups were *P. spilurus* and *P. fasciatus*, which were diverged by 9.5% net nucleotide distances under K2P model. In the genus *Nipponocypris*, both *N. temminckii* and *N. sieboldii* were found not to be monophyletic, and interspecific nucleotide diversity was 11.3% under K2P model.

In the genus *Zacco*, six separate haplogroups were observed which diverged by 3.3%–7.4% net nucleotide distance under the K2P model. One haplogroup included samples from Yong River in Chinese Zhejiang Province, the type locality of *Z. acanthogenys* and Xin'an River in Chinese Anhui Province. Therefore, this haplogroup was identified as *Z. acanthogenys*. The other five distinct haplogroups were diagnosed as *Z. platypus* under its current definition. Among them, two haplogroups were exclusively sampled in Japan, the type locality of this species, and were provisionally referred as *Z. playtpus *1–2 in this study. The rest of the three haplogroups which were distributed in China were provisionally assigned as *Zacco* sp. A–C, respectively.

In genus *Opsariichthys*, two main clades were detected consisting of 20 haplogroups and these haplogroups differed by 3.4%–15.7% net nucleotide distance under the K2P model. *Opsariichthys* one consisted of six deeply rooted haplogroups. In this clade, one haplogroup was from Japan, the type locality of *O. uncirostris*. Therefore, this haplogroup was considered as *O. uncirostris*. The remaining 5 haplogroups, which occurred in China, were ascribed to represent *O. bidens* under its current definition. One haplogroup was from the Yangtze River, the type locality of *O. bidens *and Pearl River and some rivers of the southeast coastal areas. Another haplogroup was sampled from north China such as the Amur River, the type locality of *O. uncirostris amurensis*, Tumen River, and Huai River. Depending on the number of scales, Yang and Huang (1964) suggested that *O. uncirostris amurensis* and *O. uncirostris bidens* are two subspecies; however, Chen (1982) disagreed with this suggestion. In Chen's opinion, the number of scales shows a gradual decreasing trend from north to south, which may be related to temperature difference, suggesting only one species in China, *O. bidens*. However, in the present study, we considered the two haplogroup to represent distinct species. By the type localities of the species, these two haplogroups were suggested as *O. bidens* and *O. uncirostris amurensis*. The remaining three haplogroups were from Huang River, Qiantang River, and Lijiang (a tributary of Pearl River). They were labeled as *O. uncirostris *1–3, respectively.

In *Opsariichthys* 2, there were 6 monophyletic groups with high bootstrap support representing six distinct species: *O. chengtui* from Chinese Sichuan Province, *O. duchuunguyeni *from Vietnam and Chinese Guangxi Province, *O. pachycephalus* and *O. kaopingensis *sampled in Chinese Taiwan, *O. minutus* from the upper Pearl River, and *O. hainanensis *from the south of Pearl River, Chinese Yunnan and Hainan provinces. The rest of the haplotypes sampled from China composed of eight deeply divergent haplogroups, with deep divergence (4.5%–8.4%). Perdices et al. (2004), Perdices et al. (2005), Perdices and Coelho (2006) and Zheng et al. (2016) initially erroneously identified them as *Z. platypus*, and Lin et al. (2016) identified them as *O. evolans* by mistake. However, Chen et al. (2009) considered that the samples from northern Taiwan and eastern China should be *O. evolans*, and the samples from southern China should be *O. acutipinnis*. The first haplogroup is distributed in Chinese Taiwan, the type locality of *O. evolans* and the freshwaters of the southeastern coastal China, and here, we suggested it as *O. evolans*. The next haplogroup occurring in Taipinghu, the downstream of the Yangtze River in Anhui Province, was labeled as *Opsariichthys* sp. A. The haplogroup from the middle and lower reaches of Yangtze River, the type locality of *O. acutipinnis*, the Pearl River, and Hong Kong, was suggested as *O. acutipinnis* for this haplogroup. The fourth haplogroup only distributed in Min River is provisionally designated as *Opsariichthys* sp. B. The remaining four haplogroups were all distributed in Yangtze River, but in different tributaries. Based on the molecular data and morphological characters, we proposed those samples from upper Yangtze River, the type locality of *O. macrolepis*, as a valid species (see below), and designated as *O. macrolepis*, while the others haplogroups were labeled provisionally as *Opsariichthys* sp. C–E, respectively.

### 3.2 Diagnose of species by DNA taxonomy

We designed three datasets of sequences based on the phylogenetic analysis and morphological characterization, which were the *Candidia*‐*Parazacco*‐*Nipponocypris* complex, the genus *Zacco* and the genus *Opsariichthys* to investigate the possible species in the opsariichthine. Two kinds of species delimitation tools, ABGD and PTP, were applied to construct molecular operational taxonomic units (MOTUs) for the opsariichthine specimens sequenced with cyt *b* gene, and the analysis was run for each of the datasets.

ABGD uses several priori thresholds (prior intraspecific values, P) to propose partitions of samples into MOTUs based on the distribution of pairwise genetic distances. The numbers of MOTUs defined with the ABGD method with the different prior intraspecific values from 0.001 to 0.0215 in each dataset were provided in Table 2. Sequences in *Candidia*‐*Parazacco*‐*Nipponocypris* complex dataset were analyzed with ABGD tool that resulted in a stable MOTUs count (9) for both initial partition and recursive partition for three distance metrics (JC69, K2P, p‐distance). In genus *Opsariichthys* and *Zacco* dataset, the count of MOTUs varied from 17 to 48 and 2 to 18, respectively, in addition to the partition with the 20 MOTUs in genus *Opsariichthys* and 6 MOTUs in genus *Zacco* obtained in phylogenetic analysis. In order to avoid over splitting the species, we chose to keep the smallest number of entities. So through automatic gap determination using the ABGD algorithm of, the present sequence dataset was found to comprise nine MOTUs in *Candidia*‐*Parazacco*‐*Nipponocypris* complex, 17 in genus *Opsariichthys,* and two in genus *Zacco*. The maximum likelihood identification using PTP approach was different from other methods used for taxonomic delineation. The ML analysis produced 10 MOUTs in *Candidia*‐*Parazacco*‐*Nipponocypris* complex dataset, 29 MOTUs in genus *Opsariichthys,* and 10 MOTUs in genus *Zacco*. The clustering of MOTUs obtained by the ABGD and PTP analyses is shown in Figure 2.

**Table 2:** Results of the Automatic Barcode Gap Discovery (ABGD) analyses of each dataset

Dataset	Model	X	Partition	Prior intraspecific divergence (*P*)						
				0.0215	0.0129	0.0077	0.0046	0.0028	0.0017	0.001
*Candidia*‐ *Parazacco*‐ *Nipponocypris*	Simple	1.5	Initial	9	9	9	9	9	9	9
			Recursive	9	9	9	9	9	9	9
	JC	1.5	Initial	9	9	9	9	9	9	9
			Recursive	9	9	9	9	9	9	9
	K2P	1.5	Initial	9	9	9	9	9	9	9
			Recursive	9	9	9	9	9	9	9
*Opsariichthys*	Simple	1.5	Initial	–	17	17	17	23	23	23
			Recursive	–	17	17	17	25	25	25
	JC	1.5	Initial	–	17	17	22	26	44	44
			Recursive	–	17	17	20	26	47	47
	K2P	1.5	Initial	–	17	19	20	34	44	48
			Recursive	–	17	19	20	34	43	48
*Zacco*	Simple	1.5	Initial	2	2	2	6	6	6	6
			Recursive	2	2	2	6	7	8	8
	JC	1.5	Initial	2	2	5	7	7	7	7
			Recursive	2	3	7	8	8	13	17
	K2P	1.5	Initial	2	2	5	6	7	7	7
			Recursive	2	2	7	8	8	11	18

Notes

JC69, Jukes–Cantor substitution model; K2P, Kimura 2‐parameter substitution model; simple, p‐distance; X, relative gap width.

### 3.3 Revalidation of *Opsariichthys macrolepis*

**Figure 3:**
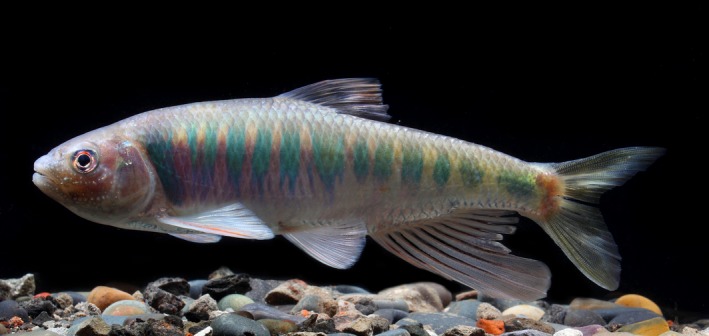
*Opsariichthys macrolepis*, alive male, IHCAS 17051383, 130.0 mm SL, Yangtze River, Hejiang Country, Sichuan Province

Based on phylogenetic analyses, the samples from the upper Yangtze River formed one haplogroup which diverged from other congeners in genus *Opsariichthys* by 3.3%–7.4% net nucleotide distance under the K2P model, and the same result was produced by DNA taxonomy tools. We suggested it should be a separate species named *Opsariichthys macrolepis*. *Z. macrolepis* was first described by Yang and Huang in 1964 as having two rows of pharyngeal teeth as the main character differing from other congeners. Then, it was treated as a synonym of *Z. platypus* by Chen (1982) because he suggested the number of rows of pharyngeal teeth was not stable. However, 150 samples of pharyngeal teeth were counted by our team before, and they all have two rows. In this study, we suggested that *Z. macrolepis* should be a valid species (Figure 3) and based on the result of the phylogenetic analysis, it belong to the genus *Opsariichthys*.

*Opsariichthys macrolepis* is distinguished from its congeners by the following unique combination of features: absence of anterior notch on upper jaw; lateral line sales 46–49; predorsal scales 17–19; scales above lateral line 8; scales below lateral line 3; scales surrounding caudal peduncle 17–18; gill rakers 8; pharyngeal teeth arranged into two rows; end of pectoral fin not reaching or slightly extending to origin of ventral fin; maxillary not reaching to or slightly extending the vertical of anterior margin of orbit; rounded tubercles on lower jaw rather small and arranging in 2–3 rows in male. The morphological descriptions are shown below.

#### 3.3.1 Synonyms

- *Zacco macrolepis*; Yang et Hwang, 1964:46 (type locality: Yangtze River)
- *Zacco macrolepis*; Bǎnǎrescu, 1968:308 (Szechwan)
- *Zacco platypus*; Chen, 1998:41 (Szechwan and Guizhou)
- *Zacco platypus*; Perdices, 2004 (the upper Yangtze River)
- *Opsariichthys evolans*; Lin, 2016 (the upper Yangtze River)

#### 3.3.2 Description

Meristic and morphometric data are listed in Table S2. Body is elongated and compressed laterally with standard length: 3.50–4.61 of depth, 3.63–4.24 of head length, and 4.83–5.91 of caudal peduncle length; head length: 3.81–4.34 of eye length, 2.67–3.34 of snout, 2.68–3.43 of interorbital width; body depth is slightly longer than head length. Dorsal III 7 is a little closer to the caudal base than to the snout; pectoral I 14 is nearly as long as head; ventral I is 7–8; anal III is 9. Anal fin extends beyond the caudal base, and the third branched ray is the longest. During the spawning season, the male develops a series of well separated and rounded horny tubercles on the snout, cheeks, and anal fin; the area below lower jaw is with two or three rows of 15–21 tubercles in total, cheek is with four longitudinal rows of tubercles, the lower two rows are separated and located at the lower cheek while the upper two rows are positioned just below the eye and the top row may or may not be interrupted; opercle is only with five small tubercles. In nuptial males, the body has 11 to 13 greenish blue stripes on the flanks, a greenish yellow caudal peduncle, and a blackish purple snout and cheek. However, the color faded when soaked in formalin.

We made comparison between *O. macrolepis* and its congeners in the Yangtze River and nearby geographic regions (Table 3). The key characters of the *O. macrolepis* that can be distinguished from *O. acutipinnis* and *O. evolans *are the following: lateral line sales 46–49 (vs. 42–43 and vs. 44–48), predorsal scales 17–19 (vs. both 15–17), scales above lateral line 8 (vs. 9 and same 8), scales below lateral line 3 (vs. both 4), scales surrounding caudal peduncle 17–18 (vs. 18–20 and vs. 16–17), the number of branched rays of pectoral 14 (vs. 15 and same 14), pectoral not reaching or slightly extending origin of ventral fin (vs. never reaching and vs. extending far beyond), maxillary not reaching to or slightly extending the vertical of anterior margin of orbit (vs. extending and vs. never extending), and rounded tubercles on lower jaw rather small and arranging as 2–3 row in male (vs. both 1 row).

**Table 3:** Morphological differences among three *Opsariichthys* species

Character	*O. macrolepis*	*O. acutipinnis*	*O. evolans*
Lateral line sales	46–49	42–44	> 45
Predorsal scales	7–19	15–17	15–17
Scales above lateral‐line	8	9	8
Scales below lateral‐line	3	4	4
Scales surrounding caudal peduncle	17–18	18–20	16–17
The number of branched rays of pectoral	14	15	14
Whether the pectoral extending the origin of ventral fin	Not reaching or slightly extending	Never reaching	Extending far beyond
Whether the maxillary extending the vertical of anterior margin of orbit	Not reaching to or slightly extending	Extending	Never extending
The number of stripes on the flanks	11–14	9–10	11
The number of tubercles on lower jaw	2–3 rows, 15–21 tubercles	1 row, <10 tubercles	1 row, 6 tubercles
Population distribution	The upper Yangtze River	Southern China	The southeast coastal areas of China
Type locality	The upper Yangtze River	Yangtze River	Chinese Taiwan

## 4 DISCUSSION

### 4.1 High species diversity in the opsariichthine group

Cryptic species refers to a group of species that have been classified with the same name or a group of morphologically indistinguishable species (Struck et al., 2018). Many cryptic species with the same morphological characters may be recognized using molecular tools and integrative taxonomy, which enrich species diversity but challenge traditional morphological taxonomy (Bickford et al., 2007). Therefore, it is necessary to increase the number of tools and characters to accurately diagnose.

At present, 23 species are suggested in the opsariichthine group, but approximately 50 species names are available and have been used (Froese & Pauly, 2018). Species of this group continues to be discovered and described. The morphological features that delineate species in opsariichthine are insufficient to describe its actual species diversity because many morphological traits can be subtle or ambiguous, which often makes it difficult to recognize and describe new species. In the present study, with more samples, based on mitochondrial cyt *b* sequences, and with the DNA delimitation approaches such as ABGD and PTP, we revised the opsariichthine group.

Our phylogenetic relationships within the opsariichthine group were mostly concordant with previous studies (Huang, Wang, & Wang, 2017), and three main groups were found: the *Candidia*‐*Parazacco*‐*Nipponocypris* group with one longitudinal stripe on the flanks, the *Zacco *group with an indistinct vertical stripe or band on the side of the body, and the *Opsariichthys* group with several distinct vertical stripes or bands along its body. Earlier studies proposed that the levels of mitochondrial DNA sequence diversity play a prominent role in taxonomy, with divergence thresholds of approximately 3% widely being accepted as indicative of species differences (Chappell et al., 2011; Hebert et al., 2003; Johnston et al., 2011). The present results showed that, when a 3% genetic distance was used as a threshold for species delimitation, there were 35 haplogroups in the opsariichthine mitochondrial phylogeny, nine haplogroups in *Candidia*‐*Parazacco*‐*Nipponocypris* group, six haplogroups in the *Zacco *group, and 20 haplogroups in the *Opsariichthys* group, respectively. We consider all of them to be putative, distinct species. In addition, the ABGD method revealed nine MOTUs in *Candidia*‐*Parazacco*‐*Nipponocypris* group, two MOTUs in the *Zacco *group, and 17 MOTUs in the *Opsariichthys* group. And the PTP method revealed 10 MOTUs in *Candidia*‐*Parazacco*‐*Nipponocypris* group, 10 MOTUs in the *Zacco *group, and 29 MOTUs in the *Opsariichthys* group. Even though the results of the three methods were not identical, all of them showed that there should be more valid species in the opsariichthine group than the present description.

The genus *Candidia* and *Candidia barbata* were treated as endemic to Chinese Taiwan by Chen (1982), based primarily on the presence of a minute barbel at the corner of the mouth. Then, Chen, Wu et al. (2008) proposed a new species *C. pingtunensis* belonging to genus *Candidia*. Wang et al. (2011) examined the phylogeographical patterns of *C. barbata* based on cyt *b* sequences and discovered six lineages. In spite of highly differentiated mtDNA, they suggested that different lineages should be considered as a single species. In our present study, the same sequences from Wang et al. (2011) were used, and the results showed that there were five haplogroups in genus *Candidia* under the methods of genetic distance threshold and ABGD, and six haplogroups revealed by PTP. Cryptic species within genus *Candidia* may explain these divergent haplogroups. The genus *Parazacco*, typified by *P. spilurus*, was described by Chen (1982) based on the presence of a postventral keel and two subspecies currently exist: *P. spilurus spilurus *and *P. spilurus fasciatus*. Kottelat (2013) upgraded them to valid species, *P. spilurus* and *P. fasciatus*. Our present results were in agreement with the latter suggestion. Nipponocypris was described by Chen, Wu et al. (2008), with both maxillary barbels and ventral keel absence, and this genus includes three valid species: two described from Japan “*N. temminckii*” and “*N. sieboldii,*” and one from Korea “*N. koreanus.*” Two species were found in this study, *N. temminckii* and *N. sieboldii*, and they were found not to be monophyletic.

*Zacco platypus* was first described with the name *Leuciscus platypus* in Japan in 1846 (Temminck & Schelegel, 1846). In 1902, Jordan and Evermann (1902) established a new genus *Zacco* using *platypu*s as the type. Later, most samples from East Asia were identified as this species (Perdices & Coelho, 2006; Perdices et al., 2004, 2005; Zheng et al., 2016). In the revision of the genera *Zacco* and *Opsariichthys*, Chen (1982) put forward four species in genus Zacco: *Z. platypus*, *Z. temmincki*, *Z. chengtui*, and *Z. taiwanensis*. The same classification results were accepted by Chen and Chu (1998). However, Chen, Wu et al. (2008) suggested that the genus *Zacco* should be limited to *Zacco platypus* based on molecular phylogenetic analysis and it may contain about 2–3 very closely related species in Chinese waters. The characteristics of the genus were recently suggested as with indistinct vertical stripes (Huang et al., 2017). Besides this, the genus also has unique type of pearl organ: on the lower cheek, and along the lower limb of the pre‐opercular, there is a row of strongly developed tubercles fused together. Our present results showed that *Z. platypus* is only distributed in Japan, and the samples from Asian continent should be different species. It is worth noting that our phylogenetic analysis showed that there are some *Zacco* species distributed in China, such as the *Z. acanhtogenys*, the species revalidated by Yin et al. (2015) under the results of molecular and morphological analysis, and some undescribed species. Besides, in the phylogenetic tree, the formerly misidentified Asian continent *Z. platypus* samples were actually in the genus *Opsariichthys*. Since the genus *Zacco* was restricted to *Z. platypus* and the related species, we agree to Chen, Huang et al. (2008) that the genus *Opsariichthys* should be enlarged to encompass these misplaced species.

According to previous studies, *Opsariichthys* with either one species, the type species *O. uncirostris* in Japan (Bǎnǎrescu, 1968), or two species, *O. uncirostris* and *O. bidens* (Chen, 1982) occured in the same geographic range as *Zacco*, except Chinese Taiwan, with the presence of a mouth with a large gape and undulated jaws as the diagnostic key features. Then four new species of *Opariichthys* have been described since 1987 from Vietnam, *O. bea*, *O. hieni*, *O. dienbienensis,* and *O. songmaensis* (Nguyen, 1987; Nguyen & Nguyen, 2000). Recently, Chen and his team conducted several morphological and molecular studies to propose new taxonomy of *Opsariichthys* (Chen & Chang, 2005; Chen, Huang et al., 2008; Chen et al., 2009; Huynh & Chen, 2013). In their study, (a) two new species were described, *Opsariithchys duchuunguyeni* and *Opsariithchys kaopingensis*, (b) four species were revalidated, *O. minutes*, *O. hainanensis*, *Z. evolans,* and *Z. acutipinnis*, were suggested as members of *Opsariichthys*, (c) *Z. taiwanensis* was treated as a synonym of *Z. pachycephalus*, and also as a member of *Opsariichthys*, (d) *Opsariichthys heini* and *Opsariichthys bea *were in fact unrelated to *Opsariichthys*. According to Chen, Huang et al. (2008), there were 12 species in genus *Opsariichthys*: *O. acutipinnis*, *O. bidens*, *O. chengtui*, *O. dienbienensis*, *O. duchuunguyeni*, *O. evolans*, *O. hainanensis*, *O. kaopingensis*, *O. minutes*, *O. pachycephalus*, *O. songmaensis,* and *O. uncirostris*. In addition, the presence of the anterior notch on upper lip was no longer as a diagnostic key feature in genus *Opsariichthys*. In the present study, 10 species were included and successfully distinguished between closely related species. Our results showed that the widely distributed species such as *O. bidens *may consist of several separate, geographically independent haplogroups, and they may represent new species. Currently available information indicates that eight haplogroups were sampled from Yangtze River, *O. acutipinnis*, *O. bidens*, *O. chengtui*, *O. macrolepis*, *Opsariichthys* sp. A, *Opsariichthys* sp. C–E, suggested that the species diversity in Yangtze River has been underestimated. Species delimitation requires additional sampling in other geographic areas and an examination of diagnostic trait. The evidence presented above suggests that species diversity within opsariichthine has been underestimated and warrants comprehensive revisions, our results call for further taxonomic studies to aid the identification of morphological or other traits useful in diagnosing opsariichthine haplogroups. Furthermore, we also conclude that the use of single mtDNA gene as molecular maker is limited in its utility. In order to confirm the results reliability, more loci, especially nuclear, should be used in the future.

### 4.2 Revalidation of the *O. macrolepis*

*O. macrolepis* was first recognized by having two rows of pharyngeal teeth by Yang and Huang (1964), and assigned in the genus *Zacco*. Later, in the revision of the genera *Zacco* and *Opsariichthys*, Bǎnǎrescu accepted the revision (1968). Nevertheless, this species was treated as a junior synonym of *Z. platypus* by Chen (1982) because he collected more samples from different locations and the results showed that the number of pharyngeal tooth rows is not stable in opsariichthine group. In our study, we found a unique lineage from the upper Yangtze River, and their morphological characters were consistent with the description of *Z. macrolepis*. And pharyngeal teeth of 150 specimens were counted by our team, they all had two rows. In our opinion, the samples collected by Chen (1982) possibly belonged to different species. Therefore, *Z. macrolepis* is considered to be a valid species, and based on the result of the phylogenetic analysis, it was suggested as a member of *Opsariichthys*.

## CONFLICT OF INTEREST

None declared.

## AUTHOR CONTRIBUTIONS

Xue Wang contributed to sampling, molecular experiment, data analyses, and writing the manuscript. Fei Liu and Dan Yu contributed to sampling and data analyses. Huanzhang Liu contributed to research design and writing the manuscript.

## Data Availability

DNA sequences have been deposited in GenBank under the accession nos. MH350437 ‐ MH350807. Details regarding individual samples are available in Supporting Information Table S1.
